# Validation Study of Diabetes Definitions Using Japanese Diagnosis Procedure Combination Data Among Hospitalized Patients

**DOI:** 10.2188/jea.JE20210024

**Published:** 2023-04-05

**Authors:** Rieko Kanehara, Atsushi Goto, Maki Goto, Toshiaki Takahashi, Motoki Iwasaki, Mitsuhiko Noda, Hikaru Ihira, Shoichiro Tsugane, Norie Sawada

**Affiliations:** 1 Epidemiology and Prevention Group, Center for Public Health Sciences, National Cancer Center, Tokyo, Japan; 2 Department of Food and Nutritional Science, Graduate School of Agriculture, Tokyo University of Agriculture, Tokyo, Japan; 3 Department of Health Data Science, Graduate School of Data Science, Yokohama City University, Kanagawa, Japan; 4 Division of Diabetes and Endocrinology, Department of Internal Medicine, Tokyo Yamate Medical Center, Japan Community Health Care Organization, Tokyo, Japan; 5 Department of Cardiology, Hiraka General Hospital, Akita, Japan; 6 Department of Diabetes, Metabolism and Endocrinology, Ichikawa Hospital, International University of Health and Welfare, Chiba, Japan; 7 Department of Epidemiology and Prevention, Center for Clinical Sciences, National Center for Global Health and Medicine, Tokyo, Japan

**Keywords:** DPC data, healthcare database, diabetes diagnosis, validation

## Abstract

**Background:**

Validation studies of diabetes definitions using nationwide healthcare databases are scarce. We evaluated the validity of diabetes definitions using disease codes and antidiabetic drug prescriptions in the Japanese Diagnosis Procedure Combination (DPC) data via medical chart review.

**Methods:**

We randomly selected 500 records among 15,334 patients who participated in the Japan Public Health Center-Based Prospective Study for the Next Generation in Yokote City and who had visited a general hospital in Akita between October 2011 and August 2018. Of the 500 patients, 98 were linked to DPC data; however, only 72 had sufficient information in the medical chart. Gold standard confirmation was performed by board-certified diabetologists. DPC-based diabetes definitions were based on the International Classification of Diseases, 10th Revision codes and antidiabetic prescriptions. Sensitivity, specificity, and the positive and negative predictive values (PPV and NPV, respectively) of DPC-based diabetes definitions were evaluated.

**Results:**

Of 72 patients, 23 were diagnosed with diabetes using chart review; 19 had a diabetes code, and 13 had both a diabetes code and antidiabetic prescriptions. The sensitivity, specificity, PPV, and NPV were 89.5% (95% confidence interval [CI], 66.9–98.7%), 96.2% (95% CI, 87.0–99.5%), 89.5% (95% CI, 66.9–98.7%), and 96.2% (95% CI, 87.0–99.5%), respectively, for (i) diabetes codes alone; 89.5% (95% CI, 66.9–98.7%), 94.3% (95% CI, 84.3–98.8%), 85.0% (95% CI, 62.1–96.8%), and 96.2% (95% CI, 86.8–99.5%) for (ii) diabetes codes and/or prescriptions; 68.4% (95% CI, 43.4–87.4%), 100% (95% CI, 93.3–100%), 100% (95% CI, 75.3–100%), and 89.8% (95% CI, 79.2–96.2%) for (iii) both diabetes codes and prescriptions.

**Conclusion:**

Our results suggest that DPC data can accurately identify diabetes among inpatients using (i) diabetes codes alone or (ii) diabetes codes and/or prescriptions.

## INTRODUCTION

Diabetes mellitus is a major global health issue, with 463 million diagnoses in 2019.^1^ In Japan, its prevalence was approximately 8.3 million in 2010 and is predicted to rise to 9.7 million by 2030.^2^ The Japanese flat-fee payment system for acute inpatient care (the Japanese Diagnosis Procedure Combination [DPC] payment system) was introduced in April 2003.^3^^–^^5^ DPC data include patient demographics, type of admission, disease names in Japanese, and disease codes from the International Classification of Diseases, 10th Revision (ICD-10) in the DPC Form 1 file (discharge summary), and prescriptions and medical procedures in the DPC EF file (medical treatment information). DPC Form 1 contains various diagnoses among inpatients that are not included in receipt data. DPC data have been used to investigate diabetes prognosis and clinical practice in Japan.^6^^–^^8^ The validity assessment of the diabetes definitions based on the DPC Form 1 and/or DPC EF file is essential to perform high-quality studies using such definitions (eg, studies of hospitalized diabetes patients).

Although the DPC system is a useful data source for clinical research, validation studies^9^^,^^10^ are indispensable for any further research. The findings of the only existing study^11^ that examined the validation of diabetes definitions based on DPC data disease codes suggested that disease codes of diabetes in the DPC data were suitable for medical research (sensitivity: 55.6%, specificity: 98.4%, positive predictive value [PPV]: 89.7%, and negative predictive value [NPV]: 89.9%). However, the evaluation was performed for various diagnoses, procedures, and laboratory data and was not specific to diabetes. Moreover, it was unclear whether medical history and prior blood test findings were considered in the previous study. Thus, in this validation study specialized for diabetes, we examined the validity of DPC-based diabetes definitions using disease codes and antidiabetic prescription compared with chart review by a board-certified diabetologist, in a Japan Public Health Center-Based Prospective Study for the Next Generation (JPHC-NEXT Study)^12^ subsample.

## METHODS

### Study population

**Figure 1.**
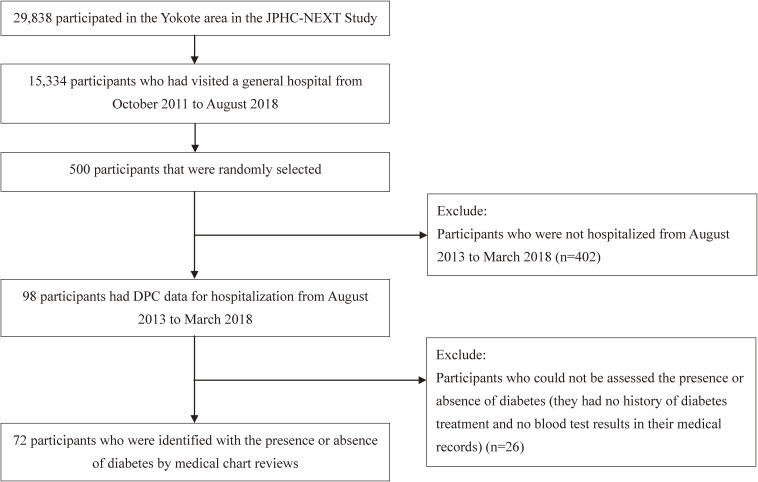
Flow chart of the study participants. DPC, the Japanese Diagnosis Procedure Combination; JPHC-NEXT, Japan Public Health Center-Based Prospective Study for the Next Generation.

The JPHC-NEXT Study was launched as a large-scale and population-based prospective cohort study in 2011.^12^ Residents who lived in seven prefectural areas (Iwate, Akita, Nagano, Ibaraki, Kochi, Ehime, and Nagasaki) and were aged 40–74 years were asked to participate in the JPHC-NEXT Study; 114,105 individuals consented.^12^ Participants from the JPHC-NEXT study who lived in Yokote City (in Akita Prefecture; *n* = 29,838) were included in this study. Among these, we included those that visited a general hospital in Yokote from October 2011 to August 2018 (*n* = 15,334, including inpatients and outpatients). The hospital (a core hospital with approximately 550 beds) administration cooperated in the JPHC-NEXT Study. In this study, data from the JPHC-NEXT Study participants’ data was linked to the patients’ data in the hospital using linkage keys (name, sex, date of birth, and zip code). For feasibility reasons, 500 out of 15,334 patients were randomly selected for a medical chart review. The chart review was projected to conduct several validation studies using a questionnaire containing diabetes history and diabetes codes on health insurance claims data and DPC data in the JPHC-NEXT Study. Of the 500 patients, 98 admission records were linked to the DPC data from August 2013 to March 2018; 402 patients were excluded because they were not hospitalized during that period. Twenty-six patients were further excluded from the main analysis because we could not assess the presence or absence of diabetes. In detail, they lacked blood test results (glycated hemoglobin [HbA1c] or blood glucose level), and we were unable to find any diabetes treatment element (including treatment at other hospitals) in their medical records. Finally, 72 patients had sufficient data to determine the presence or absence of diabetes (Figure 1). All the included participants provided written informed consent. The Institutional Review Board of the National Cancer Center of Japan approved the study (approval No. 2011-186).

### Diabetes diagnosis using medical chart review and the DPC data

We conducted the medical chart review from October 2018 to October 2019, during which a board-certified diabetologist (A. G.) reviewed each patient’s medical history, prescriptions, and laboratory findings (up to the latest results) using an electronic medical database (MegaOak, NEC, Tokyo, Japan). To identify the onset of diabetes, a medical chart review was performed using hospitalization records. Further, outpatient medical records beyond the hospitalization period were also used to ascertain the absence or presence of diabetes. Through discussions between two board-certified diabetologists (A. G. and M. G.), the presence of diabetes was assessed based on one of the following: (I) treatment history of diabetes, (II) prescription of antidiabetic agents, and (III) hyperglycemia (casual plasma glucose level ≥200 mg/dL or HbA1c level ≥6.5%).^13^ The diabetes diagnosis date was the first date satisfying the above criteria. Blood test results showing no hyperglycemia (HbA1c [<6.5%] and casual plasma glucose [<200 mg/dL]), no treatment history of diabetes, and no antidiabetic prescriptions were considered as the absence of evidence of diabetes. If both HbA1c and casual blood glucose were available, both were used to assess the lack of evidence of diabetes. If either HbA1c or casual blood glucose was unavailable, we used the available one. The period defined as no diabetes was the date of last negative blood test results. We also classified cases into types 1, 2, or steroid diabetes.

Three definitions of diabetes using the DPC data were based on the following: (i) only the diabetes codes, (ii) the diabetes codes and/or prescriptions of antidiabetic drugs, and (iii) combination of both diabetes codes and prescriptions. We used the most updated data when patients were hospitalized multiple times between August 2013 and March 2018. The six categories of disease codes using the DPC data (Form 1 file) were: “main diagnosis,” “admission-precipitating diagnosis,” “most resource-consuming diagnosis,” “second most resource-consuming diagnosis,” “comorbidities present at the time of admission,” and “conditions arising after admission”.^11^ Patients with any of the following ICD-10 codes: E10x, E11x, E12x, E13x, or E14x (that is, at least one in the disease code categories excluding “conditions arising after admission”) were considered as diabetes cases. E11x or E14x disease codes were regarded to indicate type 2 diabetes. For definitions (ii) and (iii), antidiabetic agents are listed in eTable 1. We identified the prescription records using the EF file of DPC data.

**Figure 2.**
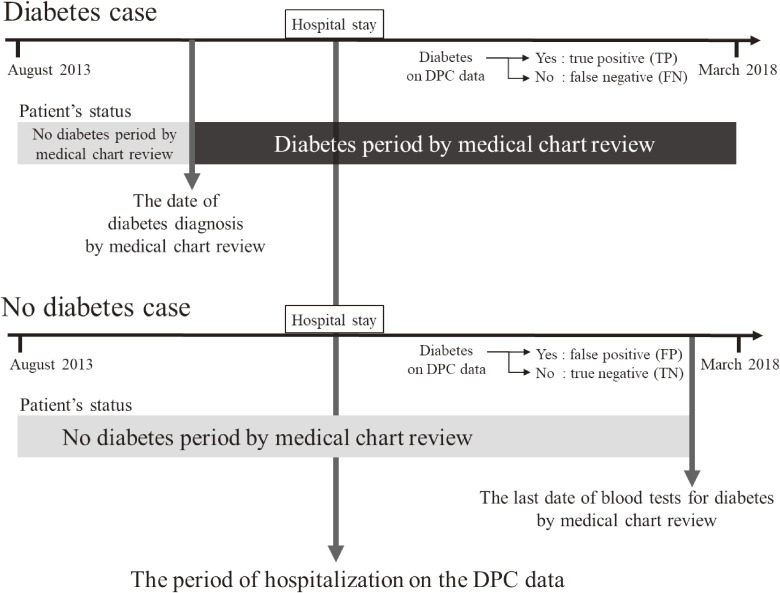
Diagram of the date of diabetes diagnosis and date of the last blood test by medical chart review and the period of hospitalization based on DPC data. DPC, Diagnosis Procedure Combination; FN, false negative; FP, false-positive; TN, true negative; TP, true positive.

The temporal context for diabetes diagnosis dates and last blood test dates for diabetes by the chart review and hospitalization period based on DPC data are shown in Figure 2.

### Statistical analysis

The means and standard deviations for age, body mass index (BMI), and admission duration using DPC data were calculated and stratified by the presence or absence of diabetes using the chart review. Similarly, the proportion of smokers (current or past), alcohol drinkers (regular or past), and occupation categories were computed. These patient characteristics were obtained from a self-administered questionnaire in the baseline survey of the JPHC-NEXT Study. Sensitivity, specificity, PPV, and NPV, and their 95% confidence intervals (CIs) for diabetes definitions using DPC data were computed. The 95% CIs were calculated using the exact binomial method.^14^ We calculated the validity indices for each of the three definitions using DPC data. We conducted two sensitivity analyses. First, we computed the validity indices using data including those of 26 patients with missing blood test results of diabetes screening and medical records of diabetes treatment. These patients were assumed not to have diabetes in the sensitivity analysis. Second, the diagnostic criterion (III) was defined as having laboratory results showing the diabetes type (twice for blood glucose levels; or once for blood glucose level and once for HbA1c level). For the diagnostic criterion (III), we regarded three patients with a single blood test as not having diabetes. Because one of the three patients hospitalized after the last date of the blood test, we could not assess the presence or absence of diabetes at the admission and excluded the patient from this sensitivity analysis. Data handling and statistical analyses were performed using Microsoft SQL Server Management Studio (Microsoft Corporation, Redmond, WA, USA) and Python 3.7.3 (Python Software Foundation, http://www.python.org).

## RESULTS

Of 72 patients, 23 (type 2 diabetes *n* = 21; steroid diabetes, *n* = 2) were diagnosed with diabetes using chart review. Patients with diabetes tended to be older, with a higher BMI, lower proportion of men and regular alcohol consumption, and higher percentage of current smokers than those without diabetes. Using the chart review, patients were assessed to have diabetes based on their history of diabetes treatment (*n* = 11), prescription of antidiabetic agents (*n* = 5), or blood test results (*n* = 7). In total, 19 patients had diabetes codes, and 13 patients had diabetes codes plus antidiabetic prescriptions in the DPC data (eTable 2).

The diabetes definition based on diabetes codes alone from the DPC data had a sensitivity, specificity, PPV, and NPV of 89.5% (95% CI, 66.9–98.7), 96.2% (95% CI, 87.0–99.5), 89.5% (95% CI, 66.9–98.7), and 96.2% (95% CI, 87.0–99.5), respectively (Table 1). For the definition of the diabetes codes and/or antidiabetic prescriptions, only one patient additionally satisfied the definition; therefore, the results were similar. However, for the definition based on both diabetes codes and antidiabetic prescriptions, sensitivity and NPV were lower (68.4% [95% CI, 43.4–87.4] and 89.8% [95% CI, 79.2–96.2]), while specificity and PPV were higher (100% [95% CI, 93.3–100] and 100% [95% CI, 75.3–100]) than for diabetes codes alone. When we computed the validity indices for type 2 diabetes only, the results were similar (Table 1).

**Table 1.** Frequencies of diabetes diagnosis and validity indices for the DPC data-based definitions

Definition on DPC data	Frequency				Sensitivity (%)	Specificity (%)	PPV (%)	NPV (%)
	TP	FN	FP	TN	(95% CI)	(95% CI)	(95% CI)	(95% CI)
All types of diabetes^a^								
(i) Diabetes codes	17	2	2	51	89.5 (66.9–98.7)	96.2 (87.0–99.5)	89.5 (66.9–98.7)	96.2 (87.0–99.5)
(ii) Diabetes codes and/or antidiabetic prescriptions	17	2	3	50	89.5 (66.9–98.7)	94.3 (84.3–98.8)	85.0 (62.1–96.8)	96.2 (86.8–99.5)
(iii) Combination of both diabetes codes and antidiabetic prescriptions	13	6	0	53	68.4 (43.4–87.4)	100 (93.3–100)	100 (75.3–100)	89.8 (79.2–96.2)

Type 2 diabetes^b^								
(i) Diabetes codes	16	1	2	51	94.1 (71.3–99.9)	96.2 (87.0–99.5)	88.9 (65.3–98.6)	98.1 (89.7–99.95)
(ii) Diabetes codes and/or antidiabetic prescriptions	16	1	3	50	94.1 (71.3–99.9)	94.3 (84.3–98.8)	84.2 (60.4–96.6)	98.0 (89.6–99.95)
(iii) Combination of both diabetes codes and antidiabetic prescriptions	13	4	0	53	76.5 (50.1–93.2)	100 (93.3–100)	100 (75.3–100)	93.0 (83.0–98.1)

CI, confidence interval; DPC, Diagnosis Procedure Combination; FN, false negative; FP, false positive; NPV, negative predictive value; PPV, positive predictive value; TN, true negative; TP, true positive.

^a^Diabetes includes all types of diabetes. International Classification of Diseases, 10^th^ revision (ICD-10) codes for DPC definition are E10x, E11x, E12x, E13x, and E14x.

^b^Diabetes includes type 2 diabetes. ICD-10 codes for DPC definition are E11x and E14x.

When we analyzed data that included those of 26 patients with missing blood test results and medical records on diabetes screening and treatment, specificity and NPV became higher because they had neither diabetes codes nor antidiabetic prescriptions in their DPC data and showed true-negative results (eTable 3). When the diagnosis criterion (III) was changed, sensitivity, specificity, PPV became slightly lower and NPV was similar compared with the results of the main analysis (eTable 4).

## DISCUSSION

For the first time, in this validation study of diabetes definitions based on DPC data among community residents, we found that the values of validity indices were sufficiently high for the following three diabetes definitions: (i) based on diabetes codes alone, (ii) based on diabetes codes and/or antidiabetic prescriptions, and (iii) based on both diabetes codes and prescriptions. Definition (i) showed a sensitivity and PPV of approximately 90% and a specificity and NPV of over 95%; two patients had a false-positive result and two had a false-negative result. With definition (iii), 0 and 6 patients showed false-positive and -negative results, respectively; definition (iii) yielded a lower sensitivity and NPV, but a higher specificity and PPV. Further, definition (ii) and (i) had a similar number of corresponding patients. Our findings suggest that DPC data can accurately define the presence or absence of diabetes among inpatients. Especially, definitions (i) and (ii) showed a satisfactory balance between sensitivity and PPV; therefore, they may be recommended for clinical research on diabetes. If researchers need a higher PPV and specificity for their study, definition (iii) may be suitable.

Compared with a previous validation study conducted among 315 patients in four hospitals on DPC-based diabetes codes,^11^ the sensitivity in our findings was higher (55.6%, 98.4%, 89.7%, and 89.9% for sensitivity, specificity, PPV, and NPV, respectively, in the previous study).^11^ Possible explanations for the difference between our findings and those of previous studies include the difference in the number of cooperating hospitals, because the accuracy of disease codes in the DPC data may vary by hospital. In the only hospital where we surveyed, the staff may be used to recording diabetes codes correctly, independent of reimbursement.

In our analysis, the judgments for up to three patients were false positives. One of those was due to inconsistency in timing of disease onset, since diabetes was not diagnosed using chart review during the hospitalization period in the DPC data but was diagnosed after the discharge. For the remaining two, the reason for the disagreement was not clear. Additionally, two patients showed false-negative results: one was receiving diabetes treatment at another hospital, while the other had steroid diabetes.

When the patients with only the results of one blood test showing the diabetic type were assessed as not having diabetes, sensitivity and PPV were approximately 90% and over 80%, respectively, for diabetes codes in the DPC data. Therefore, the diabetes definition using DPC data might accurately identify diabetes even if the diagnostic criterion (III) was defined as two tests showing the diabetes type.

The strength of this study included gold standard confirmation by board-certified diabetologists. There were certain limitations in this study. First, it was conducted at a single hospital. Our results may not represent variations in DPC data quality between hospitals in Japan, possibly limiting their generalizability. Second, the study sample size was relatively small. Third, diabetes screening was not conducted actively in the hospital where this study was performed. In fact, the presence or absence of diabetes could not be assessed in some patients. Therefore, we possibly missed potential diabetes cases and patients who received treatment for diabetes in another hospital. Fourth, the combination of linkage keys (name, sex, birth of date, and zip code) is probably less accurate than the use of unique identifiers, such as a social security number. In addition, we could not estimate a linkage rate among the current study population. Therefore, there might be linkage errors that impacted on the selection of the study population and results of the patients’ characteristics.

In conclusion, our findings suggest that the accuracy of diabetes definitions using DPC data among inpatients was high. The definition using diabetes codes alone and the definition using diabetes codes and/or antidiabetic prescriptions in DPC data may be appropriate for an investigation of diabetes. The definition using a combination of both diabetes codes and the prescriptions also can be suitable if researchers need a higher PPV and specificity. These results can facilitate the use of DPC data for clinical research on diabetes.

## SUPPLEMENTARY MATERIAL

eTable 1. Antidiabetic drugs for diabetes definition based on DPC data

eTable 2. Characteristics of the participants

eTable 3. Frequencies of diabetes diagnosis and validity indices for the DPC data-based definitions including 26 patients^a^ who lacked blood test results and any description on diabetes treatment

eTable 4. Frequencies of diabetes diagnosis and validity indices for the DPC data-based definitions changing diagnostic criterion (III) (twice for blood glucose levels; or once for blood glucose level and once for HbA1c level)
