# Supplementary material for: Validation Study of Diabetes Definitions Using Japanese Diagnosis Procedure Combination Data Among Hospitalized Patients
Source: J Epidemiol. 2023 Apr 5;33(4):165–9. doi: 10.2188/jea.JE20210024 (PMC9939922; doi:10.2188/jea.JE20210024)
Supplement: Supplementary file 1 [file je-33-165-s001.pdf]

eTable 1. Antidiabetic drugs for diabetes definition based on DPC data

Ingredients of medicine	Receipt code (unique to Japanese healthcare data)	Pharmacological subgroup of WHO ATC code	Chemical subgroup of WHO ATC code	Chemical substance of WHO ATC code	WHO ATC code
Insulin (human)	620000265, 620008897, 620008907, 620008908, 620008909, 620008932, 622114401, 640407220, 640422068, 640422074, 640453021, 640453031, 642490059, 642490107, 642490115	INSULINS AND ANALOGUES	Insulins and analogues for injection, fast-acting	Insulin (human)	A10AB01
Insulin	642490064, 642490065, 642490094, 642490095	INSULINS AND ANALOGUES	Insulins and analogues for injection, fast-acting	Insulin (beef)	A10AB02
Insulin lispro	620007460, 620008916, 622642701, 640451027, 640451028, 640451029	INSULINS AND ANALOGUES	Insulins and analogues for injection, fast-acting	Insulin lispro	A10AB04
Insulin aspart	620008893, 620008894, 620008895, 621926901, 622252701, 640451038, 640451040, 640451041	INSULINS AND ANALOGUES	Insulins and analogues for injection, fast-acting	Insulin aspart	A10AB05
Insulin glulisine	621911101, 621911201, 621911301	INSULINS AND ANALOGUES	Insulins and analogues for injection, fast-acting	Insulin glulisine	A10AB06
Insulin (human)	620000266, 620008898, 620008910, 620008911, 620008912, 620008933, 622114501, 640407221, 640412085,	INSULINS AND ANALOGUES	Insulins and analogues for	Insulin (human)	A10AC01

	640422069, 640453022, 642490061, 642490112, 642490123, 642490138		injection, intermediate-acting		
Insulin zinc	642490066, 642490071, 642490099, 642490103	INSULINS AND ANALOGUES	Insulins and analogues for injection, intermediate-acting	Insulin (beef)	A10AC02
Isophane insulin	642490063, 642490092	INSULINS AND ANALOGUES	Insulins and analogues for injection, intermediate-acting	Insulin (pork)	A10AC03
Insulin lispro	620002441, 620002444, 620007459	INSULINS AND ANALOGUES	Insulins and analogues for injection, intermediate-acting	Insulin lispro	A10AC04
Insulin (human)	620000202, 620000203, 620000204, 620000205, 620000267, 620000268, 620000269, 620000270, 620000271, 620008899, 620008913, 620008914, 620008915, 620008934, 620008935, 620008936, 622114601, 640406239, 640407222, 640412080, 640412081, 640412082, 640412083, 640412084, 640422067, 640453023, 642490121, 642490133, 642490134, 642490135, 642490136, 642490137	INSULINS AND ANALOGUES	Insulins and analogues for injection, intermediate- or long-acting combined with fast-acting	Insulin (human)	A10AD01

Insulin lispro	620002439, 620002440, 620002442, 620002443, 620007461, 620007462	INSULINS AND ANALOGUES	Insulins and analogues for injection, intermediate- or long-acting combined with fast-acting	Insulin lispro	A10AD04
Insulin aspart	620000447, 620000448, 620008896, 621973201, 621973301	INSULINS AND ANALOGUES	Insulins and analogues for injection, intermediate- or long-acting combined with fast-acting	Insulin aspart	A10AD05
Insulin degludec and Insulin aspart	622450901, 622451001	INSULINS AND ANALOGUES	Insulins and analogues for injection, intermediate- or long-acting combined with fast-acting	Insulin degludec and Insulin aspart	A10AD06
Insulin (human)	642490109, 642490125	INSULINS AND ANALOGUES	Insulins and analogues for injection, long-acting	Insulin (human)	A10AE01
Insulin zinc	642490068, 642490101	INSULINS AND ANALOGUES	Insulins and analogues for injection, long-acting	Insulin (beef)	A10AE02

Protamine insulin	642490070, 642490104	INSULINS AND ANALOGUES	Insulins and analogues for injection, long-acting	Insulin (beef)	A10AE02
Insulin glargine	620000442, 620000443, 620002445, 620004781, 620007536, 620008942, 620008943, 620008944, 620008945, 622410901, 622411001, 622440701, 622484801	INSULINS AND ANALOGUES	Insulins and analogues for injection, long-acting	Insulin glargine	A10AE04
Insulin detemir	620005900, 620005901, 620008952, 620008953, 621927001	INSULINS AND ANALOGUES	Insulins and analogues for injection, long-acting	Insulin detemir	A10AE05
Insulin degludec	622198901, 622199001	INSULINS AND ANALOGUES	Insulins and analogues for injection, long-acting	Insulin degludec	A10AE06
Metformin	610444147, 610463145, 613960009, 613960032, 620002859, 620004480, 620005570, 620009133, 621676001, 621974701, 622070801, 622242501, 622412701, 622417101, 622417201, 622421101, 622421201, 622421901, 622422001, 622424401, 622424501, 622427201, 622427301, 622432601, 622432701, 622436301, 622438401, 622438501, 622448601, 622466601	BLOOD GLUCOSE LOWERING DRUGS, EXCL. INSULINS	Biguanides	Metformin	A10BA02

Buformin	613960014, 613960015, 613960036, 620004502, 620005979, 620006872	BLOOD GLUCOSE LOWERING DRUGS, EXCL. INSULINS	Biguanides	Buformin	A10BA03
Glyclopyramide	613960011, 613960012, 620003144, 620003145	BLOOD GLUCOSE LOWERING DRUGS, EXCL. INSULINS	Sulfonylureas		A10BB
Glybuzole	613960020, 620006030	BLOOD GLUCOSE LOWERING DRUGS, EXCL. INSULINS	Sulfonylureas		A10BB
Glibenclamide	610407055, 610412056, 610433079, 610441042, 610441043, 610453088, 613960002, 613960003, 613960018, 613960019, 613960038, 613960039, 613960074, 613960076, 613960077, 613960078, 613960079, 613960080, 620000732, 620003159, 620003160, 620003604, 620006890, 620871407, 620871601, 620871907, 620872001, 620872002, 620872003, 620872004,	BLOOD GLUCOSE LOWERING DRUGS, EXCL. INSULINS	Sulfonylureas	Glibenclamide	A10BB01

	620872009, 620872016, 622009801, 622009802, 622013401, 622018801, 622018802, 622036001, 622036002, 622039901, 622075601, 622103201				
Chlorpropamide	613960006, 613960008, 613960041, 620009209	BLOOD GLUCOSE LOWERING DRUGS, EXCL. INSULINS	Sulfonylureas	Chlorpropamide	A10BB02
Tolbutamide	613960026, 613960027, 613960028, 613960050, 613960053, 613960054, 613960057, 613960058, 613960060, 613960064, 613960067, 613960068, 620003277, 620003452, 620004289, 620004842, 620006590	BLOOD GLUCOSE LOWERING DRUGS, EXCL. INSULINS	Sulfonylureas	Tolbutamide	A10BB03
Gliclazide	610406257, 610406276, 610406357, 613960037, 613960070, 613960071, 613960072, 613960073, 613960075, 620002029, 620002030, 620002031, 620002032, 620002717, 620003568, 620003661, 620003947, 620003948, 620004482, 620004580, 620006891, 620873202, 620873301, 620873402, 620873702, 622005801, 622005802,	BLOOD GLUCOSE LOWERING DRUGS, EXCL. INSULINS	Sulfonylureas	Gliclazide	A10BB09

	622141301, 622141302, 622143401, 622143402, 622169101, 622169102, 622313200				
Glimepiride	610443002, 610443003, 621982701, 621997001, 621997101, 621998701, 621998801, 621998901, 621999001, 621999301, 621999401, 621999701, 621999801, 622000601, 622000701, 622001701, 622001801, 622004701, 622004702, 622004801, 622004802, 622005501, 622005601, 622008701, 622008801, 622009901, 622010001, 622011401, 622011501, 622011601, 622011701, 622013501, 622013601, 622016001, 622016101, 622017301, 622017401, 622017501, 622017901, 622018001, 622020901, 622020903, 622021001, 622021003, 622021801, 622021901, 622022001, 622022101, 622023501, 622023601, 622025201, 622025301, 622025801, 622025901, 622026501, 622026601, 622029901, 622030001, 622031401, 622031501,	BLOOD GLUCOSE LOWERING DRUGS, EXCL. INSULINS	Sulfonylureas	Glimepiride	A10BB12

	622033001, 622033101, 622033201, 622033701, 622033801, 622035701, 622035801, 622037901, 622038001, 622038801, 622038901, 622058801, 622058901, 622059001, 622059002, 622059101, 622059102, 622088301, 622088401, 622114701, 622114801, 622118501, 622122201, 622122301, 622127301, 622127401, 622127501, 622128101, 622137701, 622141101, 622144001, 622159301, 622169301, 622171301, 622176301, 622177501, 622186201, 622187301, 622190001, 622190002, 622190801, 622193301, 622194901, 622198001, 622202201, 622202801, 622205101, 622205501, 622208901, 622211501, 622217701, 622219701, 622221001, 622222001, 622242001, 622246801, 622252501, 622254701, 622271101, 622271201, 622271301, 622313300, 622338501, 622338601, 622338701, 622631401, 622636501, 622636601				
Acetohexamide	613960017, 620000048	BLOOD GLUCOSE	Sulfonylureas	Acetohexamide	A10BB31

		LOWERING DRUGS, EXCL. INSULINS			
Sitagliptin phosphate	622625702	BLOOD GLUCOSE LOWERING DRUGS, EXCL. INSULINS	Combinations of oral blood glucose lowering drugs		A10BD
Teneligliptin hydrobromide hydrate	622573601	BLOOD GLUCOSE LOWERING DRUGS, EXCL. INSULINS	Combinations of oral blood glucose lowering drugs		A10BD
Mitiglinide calcium	622053601	BLOOD GLUCOSE LOWERING DRUGS, EXCL. INSULINS	Combinations of oral blood glucose lowering drugs		A10BD
Pioglitazone hydrochloride	621986301, 621986401	BLOOD GLUCOSE LOWERING DRUGS, EXCL. INSULINS	Combinations of oral blood glucose lowering drugs	Metformin and pioglitazone	A10BD05

Pioglitazone hydrochloride	622048401, 622048501	BLOOD GLUCOSE LOWERING DRUGS, EXCL. INSULINS	Combinations of oral blood glucose lowering drugs	Glimepiride and pioglitazone	A10BD06
Metformin and vildagliptin	622450301, 622450401	BLOOD GLUCOSE LOWERING DRUGS, EXCL. INSULINS	Combinations of oral blood glucose lowering drugs	Metformin and vildagliptin	A10BD08
Alogliptin benzoate	622086001, 622086101	BLOOD GLUCOSE LOWERING DRUGS, EXCL. INSULINS	Combinations of oral blood glucose lowering drugs	Pioglitazone and alogliptin	A10BD09
Alogliptin benzoate	622517101	BLOOD GLUCOSE LOWERING DRUGS, EXCL. INSULINS	Combinations of oral blood glucose lowering drugs	Metformin and alogliptin	A10BD13
Acarbose	613960081, 613960082, 620005359, 620005360, 620009286, 620009287, 620009288, 620009289, 620009290, 620009291, 620009292, 620009293, 620009294, 620009295, 620009296,	BLOOD GLUCOSE LOWERING DRUGS, EXCL. INSULINS	Alpha glucosidase inhibitors	Acarbose	A10BF01

	620009297, 621784902, 621785002, 621896402, 621896502, 621937101, 621937201, 621942101, 621942102, 621942201, 621942202, 621958701, 621958801, 622008501, 622008502, 622008601, 622008602, 622302201, 622302301				
Miglitol	620003127, 620003128, 620003129, 622426601, 622426701, 622432501, 622544301, 622544401, 622544501, 622560201, 622560301, 622560401, 622628601, 622628701, 622628801	BLOOD GLUCOSE LOWERING DRUGS, EXCL. INSULINS	Alpha glucosidase inhibitors	Miglitol	A10BF02
Voglibose	610406390, 610406391, 620002120, 620002121, 620002730, 620002731, 620002810, 620002811, 620002812, 620002813, 620002815, 620002816, 620002824, 620002825, 620002826, 620002827, 620002828, 620002829, 620002835, 620002836, 620002837, 620002838, 620002839, 620002840, 620002841, 620002842, 620002843, 620002844, 620002845, 620002846, 620002847, 620002848, 620004045, 620004046, 620004069, 620004070, 620004071, 620004072, 620004073,	BLOOD GLUCOSE LOWERING DRUGS, EXCL. INSULINS	Alpha glucosidase inhibitors	Voglibose	A10BF03

	620004074, 620005557, 620005558, 620005559, 620005560, 620005561, 620005562, 620005563, 620005564, 620005565, 620005566, 620006682, 620006683, 620008071, 620008072, 620008073, 620008074, 620008075, 620008076, 620008726, 620008727, 620008728, 620008729, 621665301, 621665401, 621673501, 621673601, 621683401, 621683501, 621689001, 621689101, 621689303, 621689403, 621690203, 621690303, 621690402, 621690502, 621690901, 621691001, 621691201, 621691601, 621943301, 621943401, 621953301, 621953401, 622090001, 622090101	

Pioglitazone	610432040, 610432041, 621990901, 621991001, 622041202, 622041302, 622041402, 622041502, 622042901, 622043001, 622045201, 622045301, 622045401, 622045501, 622046801, 622046901, 622047701, 622047801, 622049901, 622050001, 622053101, 622053201, 622053801, 622055801, 622055901, 622056001, 622056101, 622059201, 622059301, 622061001, 622061401, 622061501, 622061601, 622061701, 622062301, 622062302, 622062401, 622062402, 622063001, 622063101, 622063201, 622063301, 622065101, 622065201, 622065301, 622065401, 622066201, 622066301, 622071701, 622071801, 622071901, 622072001, 622078301, 622078401, 622079101, 622079201, 622081801, 622081901, 622144601, 622144701, 622147301, 622147401, 622147501, 622147601, 622155701, 622155801, 622155901, 622156001, 622156901, 622157001, 622159401, 622159501, 622163301, 622163401, 622164301,	BLOOD GLUCOSE LOWERING DRUGS, EXCL. INSULINS	Thiazolidinediones	Pioglitazone	A10BG03

	622164401, 622166801, 622166901, 622167201, 622167301, 622172101, 622172201, 622175401, 622175501, 622175601, 622175701, 622178601, 622178701, 622182401, 622182501, 622320800, 622320900			
Anagliptin	622201701	BLOOD GLUCOSE LOWERING DRUGS, EXCL. INSULINS	Dipeptidyl peptidase 4 (DPP-4) inhibitors	A10BH
Omarigliptin	622448901, 622449001	BLOOD GLUCOSE LOWERING DRUGS, EXCL. INSULINS	Dipeptidyl peptidase 4 (DPP-4) inhibitors	A10BH
Teneligliptin hydrobromide hydrate	622182601	BLOOD GLUCOSE LOWERING DRUGS, EXCL. INSULINS	Dipeptidyl peptidase 4 (DPP-4) inhibitors	A10BH

Trelagliptin succinate	622415401, 622415501	BLOOD GLUCOSE LOWERING DRUGS, EXCL. INSULINS	Dipeptidyl peptidase 4 (DPP-4) inhibitors		A10BH
Sitagliptin	621950901, 621951001, 621951101, 621970601, 621970701, 621970801, 622277501, 622288401	BLOOD GLUCOSE LOWERING DRUGS, EXCL. INSULINS	Dipeptidyl peptidase 4 (DPP-4) inhibitors	Sitagliptin	A10BH01
Vildagliptin	621980701	BLOOD GLUCOSE LOWERING DRUGS, EXCL. INSULINS	Dipeptidyl peptidase 4 (DPP-4) inhibitors	Vildagliptin	A10BH02
Saxagliptin	622245601, 622245701	BLOOD GLUCOSE LOWERING DRUGS, EXCL. INSULINS	Dipeptidyl peptidase 4 (DPP-4) inhibitors	Saxagliptin	A10BH03
Alogliptin	621986001, 621986101, 621986201	BLOOD GLUCOSE LOWERING DRUGS, EXCL. INSULINS	Dipeptidyl peptidase 4 (DPP-4) inhibitors	Alogliptin	A10BH04

Linagliptin	622093501	BLOOD GLUCOSE LOWERING DRUGS, EXCL. INSULINS	Dipeptidyl peptidase 4 (DPP-4) inhibitors	Linagliptin	A10BH05
Exenatide	622038301, 622038401, 622229001, 622406001	BLOOD GLUCOSE LOWERING DRUGS, EXCL. INSULINS	Glucagon-like peptide-1 (GLP-1) analogues	Exenatide	A10BJ01
Liraglutide	621974801	BLOOD GLUCOSE LOWERING DRUGS, EXCL. INSULINS	Glucagon-like peptide-1 (GLP-1) analogues	Liraglutide	A10BJ02
Lixisenatide	622267001	BLOOD GLUCOSE LOWERING DRUGS, EXCL. INSULINS	Glucagon-like peptide-1 (GLP-1) analogues	Lixisenatide	A10BJ03
Dulaglutide	622442201	BLOOD GLUCOSE LOWERING DRUGS, EXCL. INSULINS	Glucagon-like peptide-1 (GLP-1) analogues	Dulaglutide	A10BJ05

Ipragliflozin	622306601, 622306701	BLOOD GLUCOSE LOWERING DRUGS, EXCL. INSULINS	Sodium-glucose co-transporter 2 (SGLT2) inhibitors		A10BK
Tofogliflozin hydrate	622336801, 622340101	BLOOD GLUCOSE LOWERING DRUGS, EXCL. INSULINS	Sodium-glucose co-transporter 2 (SGLT2) inhibitors		A10BK
Luseogliflozin hydrate	622335701, 622335801	BLOOD GLUCOSE LOWERING DRUGS, EXCL. INSULINS	Sodium-glucose co-transporter 2 (SGLT2) inhibitors		A10BK
Dapagliflozin	622341901, 622342001	BLOOD GLUCOSE LOWERING DRUGS, EXCL. INSULINS	Sodium-glucose co-transporter 2 (SGLT2) inhibitors	Dapagliflozin	A10BK01
Canagliflozin	622360601	BLOOD GLUCOSE LOWERING DRUGS, EXCL. INSULINS	Sodium-glucose co-transporter 2 (SGLT2) inhibitors	Canagliflozin	A10BK02

Empagliflozin	622401201, 622401301	BLOOD GLUCOSE LOWERING DRUGS, EXCL. INSULINS	Sodium-glucose co-transporter 2 (SGLT2) inhibitors	Empagliflozin	A10BK03
Repaglinide	622040901, 622041001	BLOOD GLUCOSE LOWERING DRUGS, EXCL. INSULINS	Other blood glucose lowering drugs, excl. insulins	Repaglinide	A10BX02
Nateglinide	610432026, 610432027, 610432032, 610432033, 622119301, 622119401, 622196601, 622196701, 622230001, 622230101	BLOOD GLUCOSE LOWERING DRUGS, EXCL. INSULINS	Other blood glucose lowering drugs, excl. insulins	Nateglinide	A10BX03
Mitiglinide	620001907, 620001908, 622462401, 622462501, 622515201, 622515301, 622518101, 622518201, 622520901, 622521001, 622523301, 622523401, 622525301, 622525401	BLOOD GLUCOSE LOWERING DRUGS, EXCL. INSULINS	Other blood glucose lowering drugs, excl. insulins	Mitiglinide	A10BX08

WHO ATC code: Anatomical Therapeutic Chemical code by the World Health Organization Collaborating Centre for Drug Statistics Methodology; DPC, the Japanese Diagnosis Procedure Combination

eTable 2. Characteristics of the participants

	Diabetes (n=23)	No diabetes (n=49)	Total (n=72)
Age, years, mean (SD)[a]	67.1 (6.1)	64.9 (8.6)	65.6 (7.9)
Men, %	52.2	61.2	58.3
BMI, kg/m^2, mean (SD)	24.4(2.4)	22.7 (2.8)	23.3 (2.8)
Missing, %	4.3	2.0	2.8
Smoking, %			
Never smoker	43.5	49.0	47.2
Current smoker	26.1	16.3	19.4
Past smoker	26.1	26.5	26.4
Missing	4.3	8.2	6.9
Alcohol consumption, %			
Nondrinker or occasional drinker (< 1/week)	52.2	40.8	44.4
Regular drinker (≥ 1/week)	30.4	53.1	45.8
Past drinker	13.0	6.1	8.3
Missing	4.3	0	1.4
Occupation, %			
Agricultural or fishing work	26.1	20.4	22.2
Inoccupation	26.1	20.4	22.2
Homemaker	21.7	20.4	20.8
Professional or technical work	13.0	12.2	12.5
Service	4.3	8.2	6.9
Transportation or telecommunications	0	6.1	4.2
Administrative work	4.3	4.1	4.2

Sales	0	4.1	2.8
Others	0	4.1	2.8
Missing	4.3	0	1.4
Number of days on admission based on DPC data, mean (SD)	21 (28)	20 (33)	20 (31)
Emergency admission based on DPC data, %	56.5	49.0	51.4
Confirmed diabetes diagnosis by chart reviews, n	23	-	-
(I) History of treatments	11	-	-
(II) Prescriptions of antidiabetic agents	5	-	-
(III) Blood tests (HbA1c or blood glucose)	7	-	-
Diabetes definition based on DPC data, n			
(i) Diabetes codes	18	1	19
(ii) Diabetes codes and/or antidiabetic prescriptions	18	2	20
(iii) Combination of both diabetes codes and antidiabetic prescriptions	13	0	13

DPC, the Japanese Diagnosis Procedure Combination; HbA1c, glycated hemoglobin; SD, standard deviation.

[a] Age was that by the admission date as reported in the DPC data.

eTable 3. Frequencies of diabetes diagnosis and validity indices for the DPC data-based definitions including 26 patients[a] who lacked blood test results and any description on diabetes treatment

Definition on DPC data	Frequency TP	FN	FP	TN	Sensitivity, % (95% CI)	Specificity, % (95% CI)	PPV, % (95% CI)	NPV, % (95% CI)
All types of diabetes[b]								
(i) Diabetes codes	17	2	2	77	89.5 (66.9–98.7)	97.5 (91.2–99.7)	89.5 (66.9–98.7)	97.5 (91.2–99.7)
(ii) Diabetes codes and/or antidiabetic prescriptions	17	2	3	76	89.5 (66.9–98.7)	96.2 (89.3–99.2)	85.0 (62.1–96.8)	97.4 (91.0–99.7)
(iii) Combination of both diabetes codes and antidiabetic prescriptions	13	6	0	79	68.4 (43.4–87.4)	100 (95.4–100)	100 (75.3–100)	92.9 (85.3–97.4)

CI, confidence interval; DPC, Diagnosis Procedure Combination; FN, false negative; FP, false positive; NPV, negative predictive value; PPV, positive predictive value; TN, true negative; TP, true positive.

[a] Twenty-six patients were assumed not to have diabetes.

[b] Diabetes included all types of diabetes. International Classification of Diseases, 10th revision codes for the DPC definition are E10x, E11x, E12x, E13x, and E14x.

eTable 4. Frequencies of diabetes diagnosis and validity indices for the DPC data-based definitions changing diagnostic criterion (III) (twice for blood glucose levels; or once for blood glucose level and once for HbA1c level)

Definition on DPC data	Frequency TP	FN	FP	TN	Sensitivity, % (95% CI)	Specificity, % (95% CI)	PPV, % (95% CI)	NPV, % (95% CI)
All types of diabetes [a]								
(i) Diabetes codes	15	2	3	51	88.2 (63.6–98.5)	94.4 (84.6–98.8)	83.3 (58.6–96.4)	96.2 (87.0–99.5)
(ii) Diabetes codes and/or antidiabetic prescriptions	15	2	4	50	88.2 (63.6–98.5)	92.6 (82.1–97.9)	78.9 (54.4–93.9)	96.2 (86.8–99.5)
(iii) Combination of both diabetes codes and antidiabetic prescriptions	12	5	1	53	70.6 (44.0–89.7)	98.1 (90.1–99.95)	92.3 (64.0–99.8)	91.4 (81.0–97.1)

CI, confidence interval; DPC, Diagnosis Procedure Combination; FN, false negative; FP, false positive; HbA1c, glycated hemoglobin; NPV, negative predictive value; PPV, positive predictive value; TN, true negative; TP, true positive.

[a] Diabetes includes all types of diabetes. International Classification of Diseases, 10th revision codes for DPC definition are E10x, E11x, E12x, E13x, and E14x.
